# Supplementary material for: Real-World Biomarkers for Pediatric Takayasu Arteritis
Source: Int J Mol Sci. 2024 Jul 4;25(13):7345. doi: 10.3390/ijms25137345 (PMC11242898; doi:10.3390/ijms25137345)
Supplement: Supplementary file 1 [file ijms-25-07345-s001.zip › Suppl_material.pdf]

Supplementary material: Search strategy

Searching was performed in OVID MEDLNE, OVID EMBASE, Wiley Cochrane Library, ClinicalTrias.gov and WHO ICTRP.

The numbers of results retrieved from each source are:

- MEDLINE: 1022
- EMBASE: 1637
- Cochrane Library (CENTRAL): 40
- ClinicalTrials.gov: 27
- WHO ICTRP: 12

There are 2026 results after deduplication.

Ovid MEDLINE(R) ALL 1946 to August 14, 2023

#	Searches	Results
1	Takayasu Arteritis/	4641
2	(aortitis syndrome or pulseless disease or takayasu arteritis or takayasu disease or takayasu syndrome or takayasu's arteritis or takayasus arteritis or young female arteritides or young female arteritis or aortic arch syndrome* or anonymous artery occlusion or arteritis brachiocephalica or brachiocephalic arteritis or brachiocephalic artery occlusion or brachiocephalic ischaemia or brachiocephalic ischemia or brachiocephalic trunk occlusion or brachiocephalic vascular occlusion or innominate arterial ligation or innominate artery ligation or innominate artery occlusion or martorell syndrome or reversed coarctation or takayasu arteriopathy or takayasu ohnishi syndrome).kf,tw.	5587
3	1 or 2	6542
4	exp Child/ or exp infant/ or adolescent/ or exp pediatrics/ or child, abandoned/ or exp child, exceptional/ or child, orphaned/ or child, unwanted/ or minors/ or (pediatric* or paediatric* or child* or newborn* or congenital* or infan* or baby or babies or neonat* or pre-term or preterm* or premature birth* or NICU or preschool* or pre-school* or kindergarten* or kindergarden* or elementary school* or nursery school* or (day care* not adult*) or schoolchild* or toddler* or boy or boys or girl* or middle school* or pubescen* or juvenile* or teen* or youth* or high school* or adolesc* or pre-pubesc* or prepubesc*).mp. or (child* or adolesc* or pediat* or paediat*).jn.	5142581
5	3 and 4	1690
6	limit 5 to yr="2000 -Current"	1022
7	remove duplicates from 6	1022

Embase 1974 to 2023 August 14

#	Searches	Results
1	aortic arch syndrome/	3489
2	(aortitis syndrome or pulseless disease or takayasu arteritis or takayasu disease or takayasu syndrome or takayasu's arteritis or takayasus arteritis or young female arteritides or young female arteritis or aortic arch syndrome* or anonymous artery occlusion or arteritis brachiocephalica or brachiocephalic arteritis or brachiocephalic artery occlusion or brachiocephalic ischaemia or brachiocephalic ischemia or	7519

	brachiocephalic trunk occlusion or brachiocephalic vascular occlusion or innominate arterial ligation or innominate artery ligation or innominate artery occlusion or martorell syndrome or reversed coarctation or takayasu arteriopathy or takayasu ohnishi syndrome).kf,tw.	
3	1 or 2	8414
4	juvenile/ or exp adolescent/ or exp child/ or exp postnatal development/ or (pediatric* or paediatric* or child* or newborn* or congenital* or infan* or baby or babies or neonat* or pre term or preterm* or premature birth or NICU or preschool* or pre school* or kindergarten* or elementary school* or nursery school* or schoolchild* or toddler* or boy or boys or girl* or middle school* or pubescen* or juvenile* or teen* or youth* or high school* or adolesc* or prepubesc* or pre pubesc*).mp. or (child* or adolesc* or pediat* or paediat*).jn.	5510960
5	3 and 4	2019
6	limit 5 to yr="2000 -Current"	1663
7	remove duplicates from 6	1637

Cochrane Library

#	Search	Hits
1	[mh "Takayasu Arteritis"]	43
2	(aortitis syndrome or pulseless disease or takayasu arteritis or takayasu disease or takayasu syndrome or takayasu's arteritis or takayasus arteritis or young female arteritides or young female arteritis or aortic arch syndrome* or anonymous artery occlusion or arteritis brachiocephalica or brachiocephalic arteritis or brachiocephalic artery occlusion or brachiocephalic ischaemia or brachiocephalic ischemia or brachiocephalic trunk occlusion or brachiocephalic vascular occlusion or innominate arterial ligation or innominate artery ligation or innominate artery occlusion or martorell syndrome or reversed coarctation or takayasu arteriopathy or takayasu ohnishi syndrome):ti,ab,kw	189
3	#1 or #2	189
4	[mh "Child"] or ([mh "Congenital, Hereditary and Neonatal Diseases and Abnormalities"]) or [mh "infant"] or [mh ^"adolescent"] or [mh "pediatrics"] or [mh ^"child, abandoned"] or [mh "child, exceptional"] or [mh ^"child, orphaned"] or [mh ^"child, unwanted"] or [mh ^"minor"] or (pediatric* or paediatric* or child* or newborn* or congenital* or infan* or baby or babies or neonat* or pre-term or preterm* or (premature NEXT birth) or NICU or preschool* or (pre NEXT school*) or kindergarten* or kindergarden* or (elementary NEXT school*) or (nursery NEXT school*) or ((day NEXT care*) not adult*) or schoolchild* or toddler* or boy or boys or girl* or (middle NEXT school*) or pubescen* or juvenile* or teen* or youth* or (high NEXT school*) or adolesc* or (pre NEXT pubesc*) or prepubesc*):ti,ab,kw or (child* or adolesc* or pediat* or paediat*):so	367212
5	#3 and #4	42
6	Limit #5 to Publication Date: 2000 to 2023	40 (trials)

ClinicalTrias.gov

Condition or disease: Takayasu Arteritis
Age group: Child (birth–17)

WHO ICTRP

Takayasu Arteritis **in the Condition**
Recruitment status is ALL
Search for clinical trials in children

Supplementary material: Affected arteries in childhood TA

Involved arteries	Percentage
Abdominal aorta	53.9
Renal arteries	44.4
Thoracic aorta	33.4
Subclavian arteries	32.0
Carotid arteries	30.2
Superior mesenteric arteries	23.9
Aortic arch	19.4
Celiac trunk	18.4
Renal artery - right	15.9
Subclavian artery - left	14.5
Renal artery - left	14.5
Brachiocephalic trunk	13.5
Ascending aorta	11.7
Subclavian artery - right	10.8
Carotid artery - left	7.2
Pulmonary arteries	6.7
Aorta	5.6
Coronary arteries	5.6
Carotid artery - right	5.5
Iliac arteries	4.6
Mesenteric arteries	2.7
Inferior mesenteric artery	2.4
Vertebral arteries	2.2
Vertebral artery - right	2.1
Vertebral artery - left	2.0
Iliac artery - right	1.0
Iliac artery - left	1.0
Iliofemoral arteries	0.7
Iliofemoral artery - right	0.6
Intracranial arteries	0.4
Iliofemoral artery - left	0.4
Radial arteries	0.1
Ascending aorta and aortic arch	0.1
Axillary/brachial arteries	0.1
Femoral arteries	0.1
Femoral artery - right	0.1
Femoral artery - left	0.1
